# First Genome Assembly of the Critically Endangered Arabian Leopard (*Panthera pardus nimr*)

**DOI:** 10.3390/ijms27146115

**Published:** 2026-07-08

**Authors:** Fahad H. Alqahtani, Ion I. Măndoiu, Badr M. Al-Shomrani, Sulaiman Al-Hashmi, Fatemeh Jamshidi-Adegani, Juhaina Al-Kindi, Andrzej Golachowski, Barbara Golachowska, Abdulaziz K. Al-Jabri, Manee M. Manee

**Affiliations:** 1 National Center for Bioinformatics, King Abdulaziz City for Science and Technology, Riyadh 11442, Saudi Arabia; fqahtani@kacst.gov.sa (F.H.A.); shomrani@kacst.gov.sa (B.M.A.-S.); 2 Advanced Agricultural and Food Technologies Institute, King Abdulaziz City for Science and Technology, Riyadh 11442, Saudi Arabia; 3 School of Computing, University of Connecticut, Storrs, CT 06269, USA; ion.mandoiu@uconn.edu; 4 Laboratory for Stem Cell and Regenerative Medicine, Natural and Medical Sciences Research Center, University of Nizwa, Nizwa 616, Oman; sahashmi@unizwa.edu.om (S.A.-H.); fatemeh@unizwa.edu.om (F.J.-A.);; 5 Royal Court Affairs, Muscat 113, Oman

**Keywords:** Arabian leopard, *Panthera pardus nimr*, genome, repeats, endangered species

## Abstract

The Arabian leopard (*Panthera pardus nimr*), native to the Arabian Peninsula, is critically endangered and faces acute threats from habitat fragmentation, low population size, and genetic erosion. As a continuation of our previous study on the complete mitochondrial genome of this subspecies, we now report the first nuclear genome assembly of *P. p. nimr*, generated from the same wild-born male individual sampled in the Oman. Using PacBio HiFi long-read sequencing, we produced 162.9 gigabases (Gb) of high-fidelity data and assembled a haplotype-aware draft genome with HiFiasm. The assembly spans approximately 2.43 Gb across 94 contigs, achieving a contig N50 of 62.4 Mb and zero gap content, with BUSCO completeness of 99.4%. Genome annotation predicted 23,459 protein-coding genes with annotation BUSCO completeness of 95.0%, and 84.1% of proteins were classified as consistent with the *Panthera* lineage by OMArk. Repetitive elements occupy 34.01% of the assembly, with retroelements dominating and L1/CIN4 LINEs (15.44%) and SINEs (9.61%) representing the two largest subclasses. Comparative simple sequence repeat (SSR) analysis across six *Panthera* genomes confirmed a conserved repeat motif architecture, with no lineage-specific expansions detected in *P. p. nimr*. This nuclear genome complements the mitochondrial reference and provides a foundational resource for future studies on genetic diversity, demographic history, inbreeding load, and conservation planning for the Arabian leopard and other *Panthera* lineages.

## 1. Introduction

The Arabian leopard (*Panthera pardus nimr*) is the smallest and most geographically restricted subspecies of leopard, historically ranging across the mountainous landscapes of the Arabian Peninsula. However, its distribution has dramatically contracted in recent decades due to escalating environmental pressures and human–wildlife conflict [1,2]. Currently, fewer than 120 individuals are estimated to survive in fragmented and isolated populations, primarily in the southern part of the Oman, with possible remnant groups in Yemen, but with no evidence of the species’ continued existence in Saudi Arabia [3,4]. As a result, the subspecies is listed as Critically Endangered on the International Union for Conservation of Nature (IUCN) Red List [3]. Field surveys conducted over the past two decades have documented steep population declines and the near disappearance of individuals from previously occupied habitats [1,2,5,6]. This dramatic decline has been driven by a combination of factors, including habitat degradation, retaliatory killings in response to livestock predation, prey base depletion, and range fragmentation caused by expanding human infrastructure [6,7,8]. These pressures have not only reduced population size but have also led to genetic isolation among the remaining groups. Recent genomic analyses support this view, revealing high levels of inbreeding and markedly reduced heterozygosity, patterns that are consistent with a prolonged demographic bottleneck and indicative of an urgent need for coordinated conservation action [3,9,10].

Although conservation interventions, such as protected area expansion, captive breeding, and regional coordination, have intensified, these efforts are hindered by a limited understanding of the species’ genome-wide biology [11,12]. Most prior genetic studies of the Arabian leopard (*Panthera pardus nimr*) have relied on mitochondrial gene fragments and a small number of microsatellite loci, offering only a partial perspective on its evolutionary history and genetic health [3,12]. For instance, Al Hikmani [12] employed ND5 and CYTB sequences to demonstrate phylogenetic distinctiveness, but these markers cover only a fraction of the mitogenome. To help fill this gap, we recently assembled the first complete mitochondrial genome of a wild-born Arabian leopard from the Oman using PacBio HiFi long-read sequencing, establishing a more comprehensive foundation for maternal lineage tracing and comparative phylogenetics [9]. While this mitogenome confirmed a phylogenetic affinity with African leopards and highlighted the evolutionary distinctiveness of *P. p. nimr*, mitochondrial DNA alone represents a maternally inherited, non-recombining fraction of the genome and cannot capture autosomal diversity, patterns of inbreeding, or functional variation across the nuclear genome. Cytogenetically, the leopard has a well-characterised karyotype, with a diploid chromosome number of 2n = 38 and a fundamental number of 72 [13]. At the genomic level, chromosome-level reference assemblies are available for the African leopard and other *Panthera* species [14], including a high-altitude leopard genome [15]; however, no nuclear genome assembly had previously been reported for the Arabian leopard (*P. p. nimr*).

Whole-genome sequencing provides powerful insights into the evolutionary history, genetic architecture, and conservation status of endangered species. Genome-wide data enable detailed analyses of coding regions, structural variants, repetitive elements, runs of homozygosity, and the distribution of deleterious mutations. These features are all critical for assessing inbreeding, mutational load, and adaptive potential. In conservation contexts, such genomic information is indispensable for guiding evidence-based management strategies, including the design of genetically informed breeding programs and the selection of candidates for translocation or reintroduction. A recent study by Mochales-Riaño et al. [10] generated whole-genome sequencing data from two captive Arabian leopards and aligned the reads to a domestic cat reference genome to investigate genomic diversity, population history, and evolutionary relationships. Their analyses revealed that the Arabian leopard constitutes a distinct lineage, sister to the broader Asian clade, and has undergone a prolonged demographic decline, resulting in high levels of inbreeding and reduced heterozygosity. Despite these challenges, evidence of purging deleterious mutations was detected, offering important insights into the subspecies’ potential for long-term persistence under genetic stress. This study performed moderate-coverage whole-genome sequencing of two Arabian leopard individuals. However, de novo genome assemblies were not generated, and mapping was conducted using an existing reference sequence. To date, no nuclear genome assembly for this subspecies has been publicly released.

In this study, we address this knowledge gap by assembling the first haplotype-aware, high-fidelity draft nuclear genome of the Arabian leopard. Using PacBio HiFi long-read sequencing, we reconstructed the genome of a wild-born male individual from the Oman, the same specimen characterized in our previous mitogenome study. This high-quality draft genome provides a foundational resource for future investigations into the genetic diversity, population structure, and adaptive potential of *P. p. nimr*. In particular, it enables robust assessments of inbreeding, mutational load, and gene flow that are essential for informing evidence-based conservation management. The genome also adds to the growing collection of draft assemblies for endangered felids, thereby supporting regional and global efforts to integrate genomic data into wildlife conservation and restoration planning.

## 2. Results

### 2.1. Genome Assembly, Completeness, and Synteny Assessment

**Figure 1:**
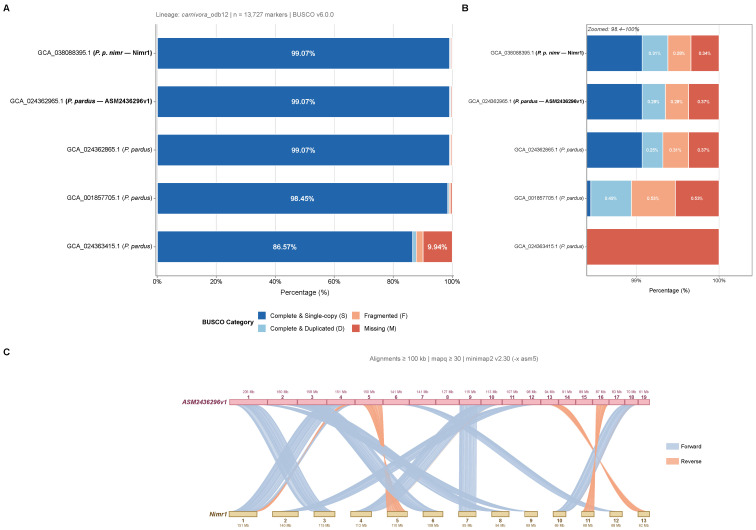
Genome assembly quality assessment and synteny analysis of five *Panthera pardus* assemblies. (**A**) BUSCO completeness assessment using the carnivora_odb12 lineage dataset (13,727 markers; BUSCO v6.0.0), showing the proportion of complete single-copy (S), complete duplicated (D), fragmented (F), and missing (M) BUSCOs for each assembly. (**B**) Zoomed view (98.4–100%) highlighting differences in the small BUSCO categories among high-quality assemblies. (**C**) Whole-genome synteny ribbon plot between the Arabian leopard assembly Nimr1 (*P. p. nimr*) and the *P. pardus* reference assembly ASM2436296v1, generated using minimap2 v2.30 (-x asm5). Ribbons connect syntenic regions (alignments ≥ 100 kb; mapping quality ≥ 30); blue ribbons indicate forward-strand collinearity and orange ribbons indicate reverse-complement (inverted) alignments. Numbers above and below bars indicate chromosome/contig rank ordered by sequence length (Mb).

The Arabian leopard nuclear genome (Nimr1; GCA_038088395.1) was assembled at the contig level, spanning ∼2.43 Gb across 94 contigs with a contig N50 of 62.4 Mbp (L50=13), N90 of 14.9 Mbp, zero gap content, and a GC content of 41.73%. Reference-based validation using QUAST against GCA_024362965.1 confirmed high concordance, with a genome fraction of 98.6%, a duplication ratio of 1.005, and a total aligned length of 2.42 Gb, with no contigs fully unaligned. Comparison with four publicly available *P. pardus* assemblies revealed that Nimr1 is among the most contiguous despite lacking Hi-C scaffolding (Table 1). GCA_024362865.1 and GCA_024362965.1, both Hi-C scaffolded, achieved scaffold N50 values of 158 Mbp and 126 Mbp, respectively. The short-read-based GCA_001857705.1 (PanPar1.0) is the most fragmented, with 50,376 scaffolds, a contig N50 of only 39 kbp, and 3.85% gap content, while GCA_024363415.1 remains a draft assembly with a scaffold N50 of 1 Mbp. Genome completeness assessed with BUSCO (carnivora_odb12; n=13,727) yielded 99.4% complete BUSCOs for Nimr1 (99.07% single-copy, 0.31% duplicated), with 0.28% fragmented and 0.34% missing, comparable to GCA_024362965.1 (99.07% single-copy) and GCA_024362865.1 (99.07% single-copy), and substantially higher than GCA_024363415.1 (87.8% complete; 9.94% missing) (Figure 1A,B). Despite its fragmentation, GCA_001857705.1 retained 98.9% completeness, indicating that short-read assemblies can capture gene space.

Whole-genome synteny analysis between Nimr1 and ASM2436296v1 (GCA_024362965.1) revealed high structural conservation across all 13 major Nimr1 contigs (≥62 Mb), each mapping collinearly to the 19 chromosome-level scaffolds of the reference (Figure 1C). The majority of alignments displayed forward-strand orientation (blue ribbons), indicative of broad synteny conservation. In contrast, a subset of reverse-complement alignments (orange ribbons) reflects localised inversions consistent with known structural variation within *Panthera* genomes. Of the 8845 total alignments generated, 7498 (84.8%) achieved the maximum mapping quality score (mapq = 60), confirming the high reliability of the observed syntenic blocks. No contigs were left unmapped, with all 94 Nimr1 contigs producing alignments against the reference.

Comparison against two additional, independently assembled chromosome-level references, leopard2_amari (GCA_024362865.1) and the high-altitude leopard genome ([15]; CNGBdb CNA0017737), recovered the same conserved collinearity, and whole-genome dotplots of all aligning contigs confirmed that individual Nimr1 contigs map uniquely to single reference scaffolds without cross-chromosomal alignments against both references (Supplementary Figures S1–S3). To assess whether the Nimr1 contigs could be confidently assigned to chromosomes, we performed a reference-guided anchoring analysis based on whole-genome alignments to the chromosome-level leopard2_amari assembly (GCA_024362865.1). Of the 82 contigs with confident alignments, 72 (88%) anchored uniquely to a single reference chromosome, each directing a mean of 95.6% of its aligned sequence to its assigned chromosome, collectively spanning 22 reference chromosomes. The remaining 10 contigs, predominantly short, showed alignments distributed across more than one reference chromosome, likely reflecting repetitive content, reference-scaffold boundaries, or genuine structural differences rather than misassembly. The per-contig anchoring summary (assignment, orientation, confidence, and conflict status) is provided in Supplementary Table S1, and the corresponding whole-genome correspondence is shown in the contig-level dotplots (Supplementary Figures S2 and S3), in which each contig aligns predominantly as a single diagonal block to one reference scaffold. Annotation of structural genomic features further supported the completeness of the assembly. barrnap identified the full set of ribosomal RNA components, including the 45S cluster genes (18S, 5.8S, and 28S) and the 5S array, confirming that the major rDNA arrays are represented. Screening for telomeric repeats with tidk, using the canonical vertebrate motif (TTAGGG)_*n*_, detected telomeric arrays at the termini of multiple contigs, including the majority of the chromosome-scale contigs (≥62 Mb), indicating that most large contigs extend to chromosome ends.

HiFiasm additionally produced two haplotype assemblies (Supplementary Table S2). Merqury assigned a consensus quality of QV 49.5 (k-mer completeness 98.03%) to the primary assembly, and 99.99% of HiFi reads mapped back at a mean coverage of 66.1×. Variant calling identified 745,564 variant sites (460,832 SNPs and 284,732 indels), of which 741,156 were heterozygous, giving a genome-wide heterozygosity of 0.30 per kb. Homozygous-block screening detected only three short blocks (F(ROH) ≈ 0), interpreted cautiously given the single individual and contig-level assembly. NCBI FCS identified a single 33-bp adaptor (removed in GCA_038088395.2) and no foreign contamination.

### 2.2. Genome Annotation

Structural gene annotation of the Nimr1 assembly was performed by integrating two complementary evidence sources: homology-based gene transfer from a conspecific reference and ab initio gene prediction. Gene models were transferred from the African leopard (*Panthera pardus pardus*; GCF_024362965.1, ASM2436296v1) to the Arabian leopard genome using Liftoff, successfully mapping 29,969 genes, including 20,318 protein-coding genes, with only 579 features left unmapped. The choice of a conspecific reference was deliberate: using the African leopard in place of the domestic cat reference reduced unmapped features from 920 to 579 and increased the number of transferred protein-coding genes from 20,120 to 20,318, reflecting the substantially higher sequence conservation within *P. pardus* subspecies compared to the inter-genus comparison with *Felis catus*. In parallel, ab initio gene prediction was performed using BRAKER3 in protein evidence mode, with the OrthoDB Vertebrata protein database (∼9.8 million proteins) provided as extrinsic evidence. BRAKER3 internally employed GeneMark-ETP for initial gene finding and AUGUSTUS for refined structural prediction, independently generating 30,379 gene models encoding 32,808 protein sequences.

**Figure 2:**
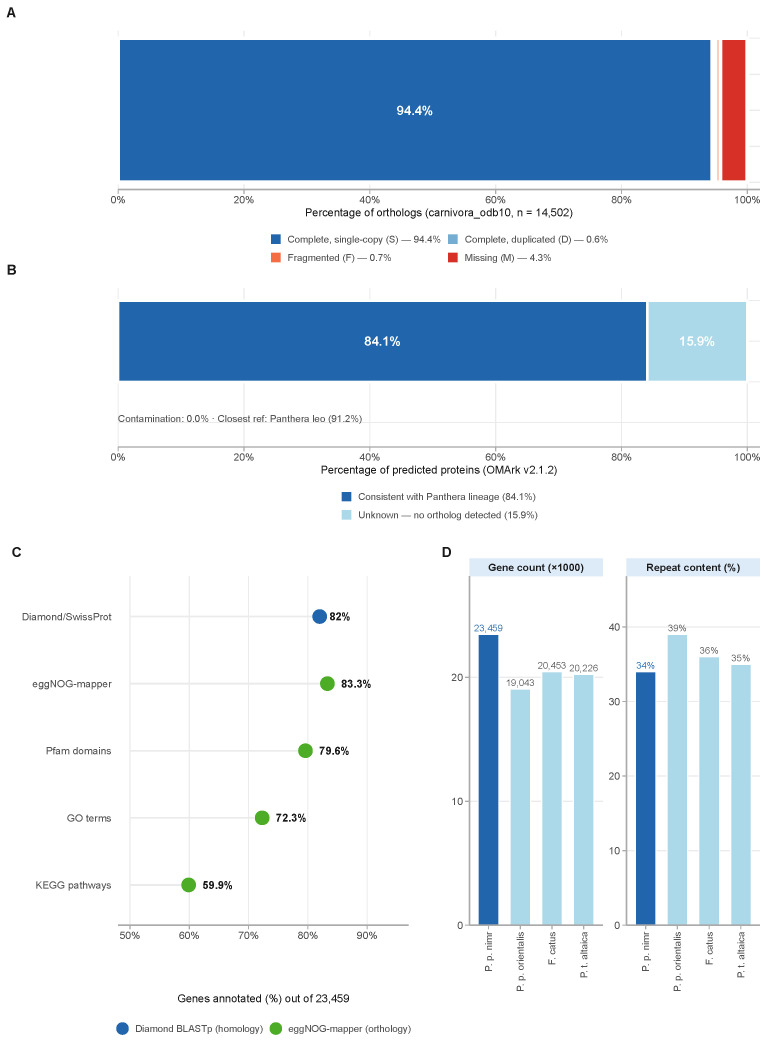
Genome annotation quality and functional characterisation of the Arabian leopard (*Panthera pardus nimr*) assembly Nimr1 (GCA_038088395.1). (**A**) BUSCO completeness assessment of the predicted proteome using the carnivora_odb10 lineage dataset (14,502 markers; BUSCO), showing the proportion of complete single-copy (S), complete duplicated (D), fragmented (F), and missing (M) orthologs. (**B**) OMArk proteome quality assessment showing the proportion of predicted proteins consistent with the *Panthera* lineage (84.1%) and proteins lacking an assignable ortholog (Unknown; 15.9%). No contamination was detected; the closest reference species was *Panthera leo* (91.2% sequence similarity). (**C**) Functional annotation coverage of the 23,459 predicted protein-coding genes across five databases: sequence homology to UniProt/Swiss-Prot via Diamond BLASTp (82.0%), orthologous group assignment via eggNOG-mapper v2.1.12 (83.3%), Pfam domain annotation (79.6%), Gene Ontology (GO) term assignment (72.3%), and KEGG pathway mapping (59.9%). (**D**) Comparison of gene content (left; protein-coding gene count $\times 10^{3}$) and repeat content (right; percentage of total assembly) between the Arabian leopard (*P. p. nimr*; this study) and three published felid genomes: Amur leopard (*P. p. orientalis*; GCF_014337955.1), domestic cat (*F. catus*; GCF_018350175.1), and Amur tiger (*P. t. altaica*; GCF_000464555.1).

Both gene sets were integrated into a non-redundant consensus using EvidenceModeler, with Liftoff predictions assigned a higher evidence weight than BRAKER3/AUGUSTUS predictions, reflecting the reliability advantage of conspecific homology transfer. The genome was partitioned into 5 Mb segments with 500 kb overlaps for parallel processing, and the resulting gene models were cleaned and validated using AGAT. The final consensus annotation yielded 23,459 protein-coding genes, with a mean of 8.2 exons per gene and a mean exon length of 179 bp. Single-exon genes accounted for 27.1% of the gene set (6347 genes), the mean CDS length was 1477 bp, the mean intron length was 4997 bp, and the total CDS length across the genome reached 34.65 Mb. The longest annotated gene spanned 2,051,392 bp, while the longest CDS extended to 65,388 bp. As shown in Figure 2D, the Arabian leopard gene count (23,459) is slightly higher than those reported for the Amur leopard (*P. p. orientalis*; 19,043 genes), the domestic cat (*Felis catus*; 20,453 genes), and the Amur tiger (*P. t. altaica*; 20,226 genes), which is expected when consensus gene sets are produced by merging multiple evidence sources through EvidenceModeler. The repeat content of the Arabian leopard genome (34%) is also lower than that of the Amur leopard (39%) and comparable to that of the domestic cat (∼36%) and the Amur tiger (∼35%) (Figure 2D).

Functional annotation was carried out using two complementary approaches (Figure 2C). Diamond BLASTp v2.1.9 was used to search predicted protein sequences against the UniProt/SwissProt database (2026_02) with an e-value threshold of $1\times 10^{-5}$, retaining the top hit per query and assigning homology-based annotations to 82% of the 23,459 predicted genes. eggNOG-mapper was run against the eggNOG database in Diamond search mode with the taxonomic scope restricted to Vertebrata, providing overall orthology-based annotations for 83.3% of predicted genes, with Gene Ontology (GO) terms assigned to 72.3%, KEGG pathway identifiers to 59.9%, and Pfam domain annotations to 79.6%. The combination of both approaches ensured that the large majority of the predicted gene set received at least one form of functional assignment.

Annotation completeness and proteome quality were evaluated independently using BUSCO and OMArk (Figure 2A,B). BUSCO analysis in protein mode against the carnivora lineage dataset (14,502 single-copy orthologs) recovered 95.0% complete genes, comprising 94.4% single-copy and 0.6% duplicated orthologs, with 0.7% fragmented and 4.3% missing, confirming high gene-space completeness consistent with the genome-level BUSCO results (Figure 2A). OMArk assessment of proteome taxonomic consistency using the OMA LUCA database classified 84.1% of predicted proteins as consistent with the expected *Panthera* lineage, with no contamination detected. The closest reference lineage identified by OMArk was *Panthera leo* (lion) at 91.2%, while the remaining 15.9% of proteins were classified as unknown, likely representing lineage-specific genes or proteins insufficiently characterized in current ortholog databases (Figure 2B). Comparison of the consensus gene set with the input predictions showed that the EVM consensus (23,459 genes) is smaller than either input (Liftoff, 29,969; BRAKER3, 30,379), indicating that EvidenceModeler consolidates rather than inflates gene models. The provenance of consensus genes and the overlap between functional–annotation sources are summarised in Supplementary Figure S4 and Supplementary Table S3.

### 2.3. Identification and Characterization of Transposable Elements

**Figure 3:**
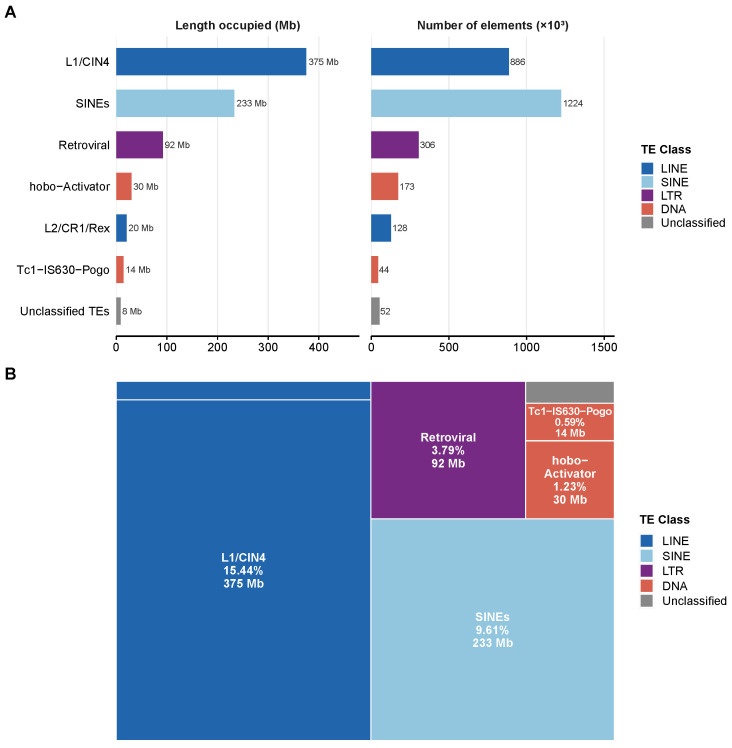
Transposable element composition of the Arabian leopard (*Panthera pardus nimr*) genome. (**A**) Abundance of each TE subclass expressed as total length occupied (Mb; left facet) and number of annotated elements (×10^3^; right facet). Bars are colour-coded by TE class: LINEs (dark blue), SINEs (light blue), LTR retrotransposons (purple), DNA transposons (orange-red), and unclassified elements (grey). Two near-zero subclasses (*Gypsy*/DIRS1, 0.0004%; other DNA transposons, 0.026%) are excluded for clarity. (**B**) Treemap in which tile area is proportional to the total base pairs occupied by each TE subclass; colour coding follows panel (**A**). Retroelements dominate the repeat landscape, with L1/CIN4 LINEs (15.44%, 375 Mb) and SINEs (9.61%, 233 Mb) representing the two most abundant TE subclasses in the genome.

Repeat annotation of the Arabian leopard (*P. pardus nimr*) genome using RepeatMasker revealed that 34.01% of the 2.43 Gb assembly is composed of repetitive elements. Transposable elements (TEs) constitute the dominant fraction of this repeat content, with retroelements alone accounting for 29.71% of the genome (721 Mb; Figure 3). Among the remaining repeat categories, simple repeats represented 1.77% (43 Mb), DNA transposons 2.18% (53 Mb), and unclassified TEs 0.35% (8 Mb), which together with the retroelement fraction (29.71%) account for the full 34.01% of masked sequence. Within the retroelement fraction, LINEs were the most abundant class, occupying 16.29% of the genome (395 Mb; 1,014,931 annotated elements). The vast majority of LINE content was attributable to the L1/CIN4 superfamily (15.44%, 375 Mb; 886,297 elements), establishing it as the single most abundant TE subclass in the Arabian leopard genome. SINEs comprised the second-largest retroelement class at 9.61% (233 Mb; 1,223,753 elements), which despite occupying less genomic space than LINEs, exceeded them in total element count, reflecting the characteristically shorter length of individual SINE copies. LTR retrotransposons contributed 3.80% of the genome (92 Mb; 308,770 elements), with the retroviral subclass (91 Mb; 305,769 elements) accounting for the near-entirety of LTR content, while *Gypsy*/DIRS1 elements were negligible (8549 bp; 78 elements).

DNA transposons represented a minor but distinct repeat fraction at 2.18% (53 Mb; 217,911 elements). The hobo-Activator superfamily was the predominant DNA transposon subclass (1.23%, 30 Mb; 173,034 elements), followed by Tc1-IS630-Pogo elements (0.59%, 14 Mb; 43,731 elements). The proportional contribution of each TE subclass to the total genome is summarised in Figure 3B, which illustrates the strong dominance of L1/CIN4 LINEs and SINEs relative to all other TE families. These two subclasses together account for 25.05% of the assembled genome sequence.

### 2.4. Identification and Characterization of Simple Sequence Repeats

**Figure 4:**
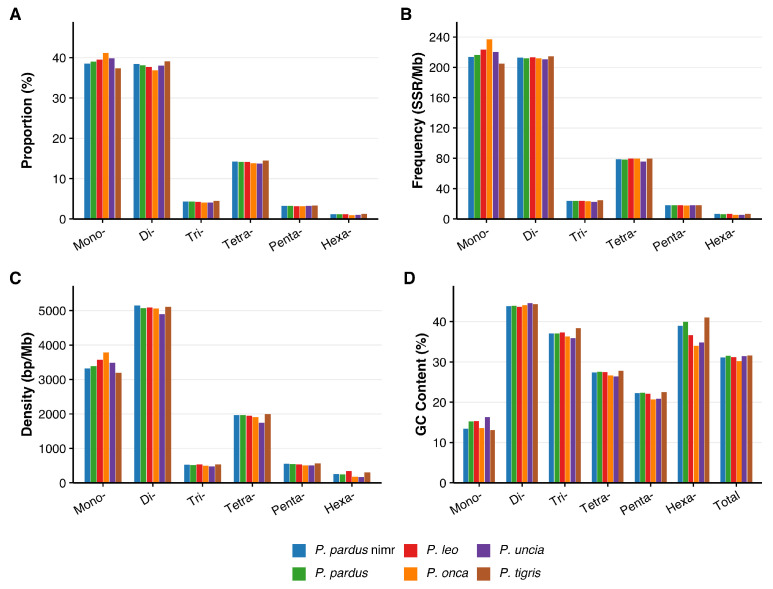
Comparative analysis of simple sequence repeat (SSR) characteristics across six *Panthera* species genomes. Four metrics are shown for each SSR repeat unit class (mono-, di-, tri-, tetra-, penta-, and hexanucleotide) across the Arabian leopard (*P. pardus nimr*), leopard (*P. pardus*), lion (*P. leo*), jaguar (*P. onca*), snow leopard (*P. uncia*), and tiger (*P. tigris*). (**A**) Proportional composition (%) of each repeat class relative to total SSR count per genome. (**B**) SSR frequency expressed as the number of SSRs per megabase (SSR/Mb) of assembled genome sequence. (**C**) SSR density expressed as the total length of SSRs per megabase (bp/Mb) of assembled genome sequence. (**D**) GC content (%) of each repeat class and of all SSRs combined (Total). All analyses were performed on perfect SSRs identified using a uniform pipeline applied to each genome assembly.

We analyzed the content of simple sequence repeats (SSRs) in the Arabian leopard genome in comparison to other *Panthera* species. This analysis contributed to a genome-wide survey of perfect SSRs across six *Panthera* species, which revealed conserved patterns in SSR type distribution, frequency, density, and GC content, along with species-specific differences (Figure 4). The SSR class distribution revealed that mononucleotide repeats were the most abundant across all genomes, including that of *P. p. nimr*, followed by di- and trinucleotide repeats, reflecting a broadly conserved pattern in SSR composition within the genus. The SSR frequency, measured as the number of SSRs per megabase, showed that the Arabian leopard genome harbors a similar repeat abundance compared to other species, with slight elevations observed in *P. tigris*. SSR density patterns, defined by the total SSR base pairs per megabase, mirrored these trends and confirmed that the Arabian leopard genome does not show major repeat expansion or contraction. Additionally, the GC content of SSRs remained relatively stable across species and repeat classes, with the Arabian leopard genome displaying values comparable to other *Panthera* species. These results collectively demonstrate a conserved SSR landscape across *Panthera*, with the Arabian leopard genome exhibiting no extreme deviations in SSR structure or composition.

The number of SSRs and their maximum repeat counts varied considerably across microsatellite types and leopard genomes, indicating substantial diversity in repeat expansion patterns (Supplementary Figure S5). Mononucleotide and dinucleotide SSRs were especially abundant and showed the widest range of repeat numbers, while tri-, tetra-, penta-, and hexanucleotide SSRs were less frequent. The overall repeat motif profiles appeared largely conserved across the six *Panthera* species, including *P. p. nimr*, though minor differences in the distribution of longer repeat tracts were observed.

Analysis of SSR motif types across six leopard genomes revealed conserved patterns of dominant motifs within each repeat class (Supplementary Figure S6). In mononucleotide SSRs, A-rich motifs were the most frequent across all species. Dinucleotide motifs were predominantly composed of (AG)_n_ repeats, while (AC)_n_ and (AT)_n_ motifs were also common but less abundant. Among trinucleotide repeats, motifs such as (AAC)_n_, (AAT)_n_, and (AGG)_n_ showed higher frequencies, with subtle variation between species. Tetranucleotide SSRs were led by (AAAT)_n_ and (AAAG)_n_ motifs, whereas pentanucleotide and hexanucleotide classes exhibited a broader diversity of low-frequency motifs, such as (AAAAC)_n_, (AAAAT)_n_, (AAAAAC)_n_, and (AGATAT)_n_. These patterns were largely consistent across *Panthera* species, including *P. pardus nimr*.

## 3. Discussion

The Arabian leopard (*Panthera pardus nimr*) is among the most imperiled felids on Earth. With fewer than 120 individuals estimated to persist in the wild, confined largely to the mountainous regions of the southern part of the Oman and possibly remnant patches in Yemen, the subspecies faces a confluence of threats that have driven it to the edge of extinction [3,4]. Habitat degradation, retaliatory killings in response to livestock predation, severe depletion of prey, and the progressive fragmentation of its range by expanding human infrastructure have collectively reduced both the size and connectivity of its remaining populations [6,7,8]. These pressures have not only diminished census numbers but have also imposed a significant genetic cost: genomic analyses have revealed elevated inbreeding coefficients, markedly reduced heterozygosity, and signatures consistent with a prolonged demographic bottleneck [3,9,10]. The resulting genetic erosion may compromise adaptive potential and long-term population viability, underscoring the urgency of conservation action informed by robust genomic data.

Despite this crisis, the genomic resources available for *P. p. nimr* have remained remarkably limited. Most prior genetic studies have relied on fragments of mitochondrial genes and a restricted panel of microsatellite loci, providing only a narrow window into the subspecies’ evolutionary history and genetic health [3,12]. Mitochondrial markers, while informative for inferring maternal lineage and broad phylogenetic placement, represent a single non-recombining locus that cannot capture the breadth of autosomal diversity, reveal patterns of inbreeding, or characterise functional variation across the nuclear genome. Although a recent study by Mochales-Riaño et al. [10] employed whole-genome sequencing of two captive Arabian leopards to investigate genomic diversity and population history, those analyses relied on alignment to an existing domestic cat reference rather than a *de novo* assembly, and no nuclear genome assembly for *P. p. nimr* was publicly available until now. This absence has been a significant impediment to conservation planning, precluding analyses of coding variation, structural genomic features, runs of homozygosity, and the distribution of deleterious mutations that are essential for evidence-based management.

The present study directly addresses this gap. Building on our earlier assembly of the first complete mitochondrial genome of a wild-born Arabian leopard [9], which confirmed phylogenetic affinity with African leopards and established *P. p. nimr* as an evolutionarily distinct lineage, we now extend that foundational resource with the first haplotype-aware nuclear genome assembly of this subspecies, generated from the same wild-born male individual sampled in the Oman. Together, the mitogenome and Nimr1 nuclear assembly constitute a comprehensive genomic reference for *P. p. nimr*, enabling a far richer suite of downstream analyses than was previously possible.

The Nimr1 assembly generated from PacBio HiFi long-read sequencing demonstrates a high level of contiguity and completeness that compares favourably with existing *P. pardus* reference genomes. The assembly spans approximately 2.43 Gb across only 94 contigs, achieving a contig N50 of 62.4 Mb, a value substantially exceeding those of most existing leopard assemblies produced with short-read or Hi-C-assisted scaffolding technologies. For instance, the widely used *P. pardus* assembly GCA_024362965.1 (ASM2436296v1), despite Hi-C scaffolding, achieves a contig N50 of only 22 Mb, while the earlier short-read-based PanPar1.0 assembly is severely fragmented, with a contig N50 of 39 kb and 3.85% gap content (Table 1). The zero gap content of Nimr1, combined with BUSCO completeness of 99.4% against the carnivora_odb12 lineage dataset (13,727 markers), confirms that the HiFi-based approach delivers a gene-complete and structurally coherent assembly even without chromosome-level scaffolding. Whole-genome synteny analysis against ASM2436296v1 further demonstrated that all 13 major Nimr1 contigs map collinearly to the 19 chromosome-level scaffolds of the reference, with 84.8% of high-confidence alignments achieving the maximum mapping quality score, confirming broad synteny conservation and the absence of major structural anomalies. These quality metrics are consistent with the high fidelity expected from PacBio HiFi reads, making them particularly well-suited for resolving repetitive regions that fragment short-read assemblies.

Genome annotation of the Nimr1 assembly predicted 23,459 protein-coding genes, somewhat higher than the 19,043 reported for the leopard (*P. pardus*) [16], 20,453 for the domestic cat (*Felis catus*; GCF_018350175.1, Figure 2D) [17], and 20,226 for the Amur tiger (*P. t. altaica*) [18], consistent with the expected variation in gene counts across annotation pipelines. Functional coverage was broad (BLASTp 82%, eggNOG 83.3%, GO 72.3%, KEGG 59.9%, Pfam 79.6%), and OMArk classified 84.1% of predicted proteins as consistent with the *Panthera* lineage, with *P. leo* identified as the closest reference at 91.2% similarity, reflecting the well-established phylogenetic proximity of lion and leopard [10]. Repetitive elements occupy 34.01% of the Nimr1 assembly, lower than the leopard (39%, [16]) and comparable to the Amur tiger (∼35%) and domestic cat (∼36%, Figure 2D); retroelements dominate at 29.71%, with L1/CIN4 LINEs (15.44%) and SINEs (9.61%) as the two largest subclasses, consistent with the conserved TE proportions documented across felids and mammals [19,20]. SSR analysis across six *Panthera* genomes revealed a conserved motif architecture, with A-rich mononucleotides (AG)_n_ and (AAC/AAT)_n_ predominating in all species, confirming that *P. p. nimr* does not harbour lineage-specific repeat expansions that might compromise genome integrity.

Beyond demographic and conservation-genetic applications, a high-quality conspecific assembly such as Nimr1 can support the development of forensic identification tools relevant to the illegal trade in endangered felids. Because seized specimens are often degraded or processed, DNA-based species and individual identification is frequently required. Whole-genome assemblies are increasingly used to design such markers, as in silico mining can identify polymorphic SSR/STR loci suitable for multiplex panels for individualisation, sex determination, and parentage assignment, alongside qPCR assays for species detection and quantitation of trace samples. This approach has been applied across the genus, including microsatellite discovery from seven *Panthera* genomes [21], a genome-derived 15-locus multiplex (TPI-plex) for pedigree identification in tigers [22], and a combined qPCR/STR system for *P. pardus* [23]. Because big-cat studies have often relied on cross-amplified domestic cat markers, a subspecies-specific reference for *P. p. nimr* may facilitate the design of Arabian leopard-tailored panels to support forensic casework and parentage management within the captive breeding programme.

The Nimr1 assembly situates *P. p. nimr* within the broader leopard genomic landscape established by Paijmans et al. [24], who documented that Asian leopard populations are characterised by stronger isolation by distance and substantially lower heterozygosity than African counterparts, consistent with a single out-of-Africa dispersal approximately 500–600 thousand years ago. The chromosome-level African leopard assemblies of Armstrong et al. [14], from which our primary (ASM2436296v1) and secondary (leopard2_amari) synteny references derive, together with the high-altitude leopard genome of Zhou et al. [15], provided the independent chromosome-scale references against which the collinearity and chromosomal correspondence of Nimr1 were validated. The geographic position of *P. p. nimr* at the southwestern margin of the Asian clade, combined with genomic evidence of high inbreeding [10] and its mitochondrial affinity with African leopards [9], reflects both this range-wide diversity gradient and a history of African allele transfer within the last 100,000 years [24]. Past gene flow with African and Anatolian (*P. p. tulliana*) leopards detected by Mochales-Riaño et al. [10] further indicates that the current genetic isolation of *P. p. nimr* is a recent consequence of habitat fragmentation rather than an ancient condition. The Nimr1 assembly now provides the conspecific reference required to quantify these patterns with greater precision than was possible using the domestic cat genome.

Several limitations of the present study warrant acknowledgment. The Nimr1 assembly is a contig-level draft that has not yet achieved chromosome-level scaffolding, precluding the assignment of the 13 major contigs to specific chromosomes and limiting downstream analyses that require chromosome-scale organisation. Comprehensive annotation of additional small non-coding RNA families and the resolution of centromeric and satellite repeat arrays similarly depend on chromosome-scale assembly data, as these elements are typically collapsed or unresolved in contig-level assemblies, and are identified as priorities for future assembly upgrades. The assembly further derives from a single individual, so population-level diversity metrics cannot be estimated directly, and the 15.9% of OMArk-unclassified proteins likely reflects a combination of lineage-specific genes and entries insufficiently represented in current ortholog databases. The haplotype assemblies represent computational phasing rather than verified paternal/maternal haplotypes, and the near-zero F(ROH) likely underestimates true autozygosity in the absence of population allele frequencies and chromosome-scale scaffolding. These constraints point directly to the priorities for future work: chromosome-level scaffolding via Hi-C or optical mapping would resolve the first limitation and bring Nimr1 into alignment with emerging reference genome standards; population-level resequencing of wild and captive individuals of known origin would enable genome-wide characterisation of diversity, inbreeding, and demographic history [10], and inform whole-genome management of the captive breeding programme, which currently maintains only a few founders [25]. Comparative analyses with other *Panthera* reference genomes will additionally enable evolutionary study of coding variation and adaptive divergence across the radiation.

## 4. Materials and Methods

### 4.1. Sample Collection and Ethical Approval

**Figure 5:**
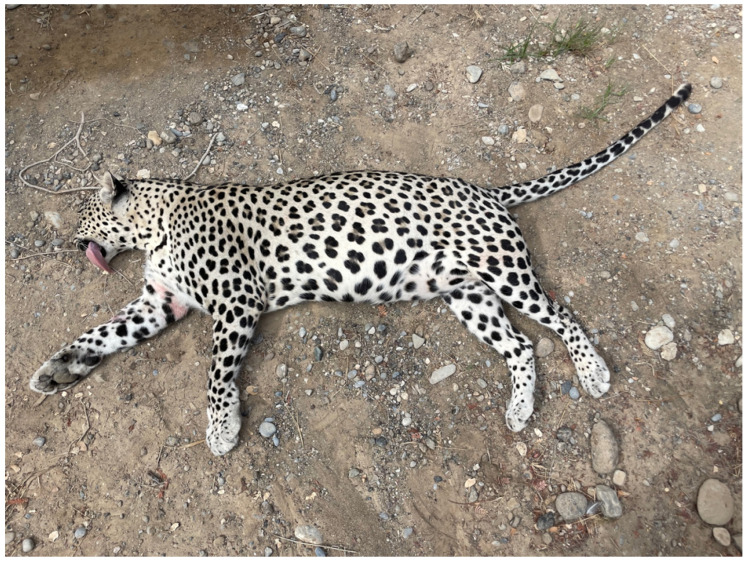
The wild-born male Arabian leopard (*Panthera pardus nimr*) sampled in this study, photographed at the Directorate General of Veterinary Services, Royal Court Affairs, Oman. Photograph by Andrzej Golachowski; originally published in Alqahtani et al. [9].

As part of routine veterinary procedures associated with relocation, a health assessment was conducted on 4 September 2023 for a male Arabian leopard (*Panthera pardus nimr*), born in 2011 and housed at the Wildlife Breeding Center in A’Seeb, Barka, in the Oman (Figure 5). The individual was scheduled for transfer to the Directorate General of Veterinary Services, Royal Court Affairs. To facilitate safe and humane handling, the leopard was chemically immobilized using a combination of tiletamine and zolazepam (Telazol, Zoetis Inc., Parsippany, NJ, USA) and medetomidine (Domitor, Orion Pharma, Espoo, Finland). A working solution was prepared by mixing 5 mL of Domitor with one vial of Telazol, yielding a concentration of 50 mg zolazepam, 50 mg tiletamine, and 1 mg medetomidine per milliliter. This was delivered via 1.5-mL dart syringes fitted with 1.50 × 30-mm collared needles using a CO_2_-powered rifle. Once anesthetized, blood samples were collected from the left jugular vein into EDTA and serum tubes. After approximately 20 min, anesthesia was reversed with intramuscular injections of 1 mL (5 mg) atipamezole (Antisedan, Orion Pharma, Espoo, Finland) and 8 mL (0.80 mg) flumazenil (Flumazenil, B. Braun Melsungen AG, Melsungen, Germany). The leopard was monitored and returned safely to its enclosure following recovery. This wild-born individual was specifically selected to represent natural genetic diversity in downstream analyses. All procedures were conducted following ethical guidelines for wildlife research, under protocol approval number VCGSR/AREC/05/2023, approved on 8 September 2023.

### 4.2. DNA Extraction

Genomic DNA was extracted from whole blood using the PureLink™ Genomic DNA Mini Kit (Thermo Fisher Scientific, Waltham, MA, USA), following the manufacturer’s instructions. A total of 200 μL of blood was combined with 20 μL each of Proteinase K and RNase A, vortexed gently, and incubated at room temperature for 2 min. Cell lysis was performed by adding 200 μL of lysis/binding buffer and incubating at 55 °C for 10 min, followed by the addition of 200 μL of ethanol. The lysate was then loaded onto a spin column, centrifuged, washed sequentially with Wash Buffers 1 and 2, and eluted in 70 μL of elution buffer at 14,000× *g*. The protocol was optimized to ensure high DNA yield and purity from a limited blood volume. DNA concentration and purity were assessed by UV absorbance at 260/280 nm, and integrity was verified by 1% agarose gel electrophoresis. The extracted DNA was stored at −20 °C and archived at the Laboratory for Stem Cells and Regenerative Medicine, University of Nizwa (voucher number: LSCRM-L-02-04, 22.91° N, 57.67° E; contact: Sulaiman Al-Hashmi, sahashmi@unizwa.edu.om).

### 4.3. Library Preparation and Sequencing

Library preparation was performed using the PacBio SMRTbell Express Template Prep Kit v3.0 (Pacific Biosciences, Menlo Park, CA, USA), following the manufacturer’s standard protocols. Sequencing was carried out on the PacBio Revio platform, selected for its capacity to generate long, high-fidelity (HiFi) reads, which are well-suited for producing a contiguous and accurate nuclear genome assembly. The sequencing was conducted by Macrogen Inc. (Seoul, Republic of Korea), yielding two datasets with a combined total of 162.90 Gbp of HiFi reads, with an average Q-score of 29. The raw reads were submitted to the NCBI Sequence Read Archive under accession number PRJNA1091853.

### 4.4. Genome Assembly and Quality Assessment

De novo genome assembly was performed using HiFiasm v0.19.8 [26] in default mode, which employs haplotype-aware error correction and produces a primary assembly alongside phased haplotype contigs (haplotype 1 and haplotype 2). HiFi reads were provided directly as input without prior trimming, as HiFiasm performs internal read correction. The primary assembly contigs were extracted from the output GFA file and converted to FASTA format using awk. Assembly contiguity and accuracy were evaluated using QUAST v5.2.0 [27] in reference-based mode, using the publicly available *P. pardus* assembly GCA_024362965.1 (ASM2436296v1) as the reference genome, to generate standard assembly statistics including total length, number of contigs, N50, L50, N90, L90, GC content, genome fraction, and duplication ratio. Genome completeness was assessed using BUSCO v6.0.0 [28] against the carnivora_odb12 lineage dataset (13,727 BUSCO markers; created 1 July 2025), the most taxonomically specific lineage available for *Panthera pardus*, run in euk_genome_min mode with miniprot v0.18 as the gene predictor. The same analysis was applied to four additional publicly available *P. pardus* assemblies (GCA_024362965.1, GCA_024362865.1, GCA_001857705.1, and GCA_024363415.1) for comparative benchmarking. BUSCO results are reported using standard notation: C (Complete), S (Single-copy), D (Duplicated), F (Fragmented), and M (Missing).

Whole-genome synteny between the Nimr1 assembly and the reference *P. pardus* genome GCA_024362965.1 (ASM2436296v1) was assessed using minimap2 v2.30 [29] with the assembly-to-assembly preset (-x asm5), which is optimised for comparing highly similar genome assemblies with a sequence divergence of approximately 0.1%. The output was generated in PAF format with the –cs flag to retain cigar-string information. Only high-confidence alignments were retained for visualisation, applying a minimum mapping quality threshold of 30 (mapq ≥ 30) and a minimum alignment length of 100 kb. The same procedure was repeated against two additional chromosome-level references, leopard2_amari (GCA_024362865.1) and the high-altitude leopard genome of Zhou et al. [15] (CNGBdb CNA0017737), and whole-genome dotplots of all aligning Nimr1 contigs against each reference were generated to assess whether contigs map uniquely to single chromosomes. Synteny was visualised as a ribbon plot displaying the 19 chromosome-level scaffolds of ASM2436296v1 and the 13 largest contigs of Nimr1 (all ≥62 Mb), with ribbons coloured by strand orientation (forward: blue; reverse complement: orange). Reference-guided contig anchoring was inferred from the minimap2 (-x asm5) alignments to the chromosome-level leopard2_amari assembly (GCA_024362865.1). For each contig, the reference chromosome receiving the largest share of aligned sequence (alignments ≥ 10 kb, mapping quality ≥ 30) was taken as the primary assignment, with orientation determined by the dominant alignment strand. Contigs directing less than 85% of their aligned length to a single reference chromosome were flagged as conflicting. Assembly accuracy and completeness were assessed with Merqury using a 21-mer database built from the HiFi reads, reporting consensus quality (QV) and k-mer completeness for the primary and both haplotype assemblies. HiFi reads were mapped to the primary assembly with minimap2 v2.30 (map-hifi preset) to obtain mapping rate and coverage. Variants were called with bcftools to estimate genome-wide heterozygosity and SNP/indel density, and homozygous blocks were screened with bcftools roh. Contamination was screened by the NCBI Foreign Contamination Screen (FCS) during GenBank submission.

### 4.5. Genome Annotation

De novo repeat identification was performed using RepeatModeler v2.0 [30] with the -LTRStruct flag enabled, executed within the Dfam TE Tools Singularity container. The resulting custom repeat library was supplied to RepeatMasker v4.2.1 [31] run with the -xsmall flag to produce a soft-masked genome in which repetitive regions are represented as lowercase characters, as required by downstream gene prediction tools. Structural gene annotation combined homology-based gene transfer and ab initio prediction. Gene models were transferred from the African leopard (*Panthera pardus pardus*; GCF_024362965.1, ASM2436296v1) to the Arabian leopard genome using Liftoff v1.6.3 [32] with the -exclude_partial flag to remove incomplete transfers; the African leopard was selected as the reference given the higher sequence conservation within *Panthera pardus* subspecies. Ab initio gene prediction was performed using BRAKER3 v3.0.8 [33] in protein evidence mode, with the OrthoDB v11 Vertebrata protein database [34] (∼9.8 million proteins) provided as extrinsic evidence; BRAKER3 internally employs GeneMark-ETP [35] for initial gene finding and AUGUSTUS v3.5.0 [36] for refined structural prediction. Gene models from both approaches were merged into a non-redundant consensus using EvidenceModeler v2.1.0 (EVM) [37], with Liftoff predictions assigned a weight of 5 and BRAKER3/AUGUSTUS predictions a weight of 2, reflecting the higher reliability of the conspecific homology transfer. The genome was partitioned into 5 Mb segments with 500 kb overlaps for parallel processing, and the resulting gene set was cleaned and validated using AGAT v0.8.0 [38].

Functional annotation was carried out using two complementary approaches: Diamond BLASTp v2.0.14 [39] was used to search predicted protein sequences against the UniProt/Swiss-Prot database [40] with an e-value threshold of $1\times 10^{-5}$, retaining the top hit per query, and eggNOG-mapper v2.1.12 [41] was run against the eggNOG v5.0.2 database [42] in Diamond search mode with –tax_scope Vertebrata and –go_evidence all to assign Gene Ontology (GO) terms, KEGG pathway identifiers, COG functional categories, and Pfam domain annotations. Annotation completeness was assessed in protein mode using BUSCO v5.2.2 [28] against the carnivora_odb10 lineage dataset (14,502 ortholog markers), the version bundled within the annotation pipeline used for proteome evaluation. Because genome-mode BUSCO (run on the assembly via miniprot) and protein-mode BUSCO (run on the final predicted proteome) quantify different stages of completeness, namely gene space present in the assembly versus gene models retained after consensus annotation, the two scores are complementary rather than directly comparable. Proteome quality and taxonomic consistency were evaluated using OMArk v2.1.2 [43] with the OMA LUCA database, which classifies predicted proteins as consistent, inconsistent, contaminant, or unknown relative to the expected taxonomic lineage. Consensus gene provenance and functional-annotation overlap were assessed by coordinate overlap (BEDTools intersect, ≥50% reciprocal) and by comparing the Diamond BLASTp and eggNOG-mapper assignments, respectively.Ribosomal RNA genes were annotated with barrnap v0.9 [44] in eukaryotic mode, and telomeric repeat arrays were identified with tidk [45] using the canonical vertebrate telomeric motif (TTAGGG)_*n*_.

### 4.6. Analysis of Simple Sequence Repeats

We selected genome assemblies from six *Panthera* species, including our *Panthera pardus nimr* (Arabian leopard) genome, as well as *Panthera pardus*, *Panthera leo*, *Panthera onca*, *Panthera uncia*, and *Panthera tigris*, for comparative analysis of simple sequence repeat (SSR) distribution across their genomes. The genome assemblies, identified by accession numbers GCA_038088395.1, GCA_024362965.1, GCA_018350215.1, GCA_028533385.1, GCA_023721935.1, and GCA_018350195.2, were retrieved in FASTA format from the NCBI GenBank Genomes FTP repository (ftp://ftp.ncbi.nlm.nih.gov/genomes/genbank/, accessed on 1 May 2026). A genome-wide scan for SSRs was conducted using the PERF software tool, version 0.2.5 [46], applied to each complete genome. Specific filtering criteria were used to detect perfect SSRs in the genome sequences. Repeats ranging from 1 to 6 nucleotides in length were targeted, with thresholds set at a minimum of 12 repeats for mononucleotides, 7 for dinucleotides, 5 for trinucleotides, and 4 for tetra-, penta-, and hexanucleotides, following established guidelines from prior studies [47,48]. SSR motifs that were circular permutations or reverse complements of each other were classified as the same repeat type for the purposes of this analysis [49,50]. SSR motif types were analyzed based on their relative frequency (count per megabase) and relative density (cumulative base pair length per megabase) across the genomes. All statistical computations and visualizations were performed using the R programming language, version 4.3.2 (R Core Team, 2023).

## 5. Conclusions

The Nimr1 assembly provides the first nuclear genome reference for *P. p. nimr*, transitioning this critically endangered subspecies from a data-poor to a genomically informed conservation framework. Together with the previously reported mitogenome [9], these resources establish a foundation for characterising genetic diversity, inbreeding load, demographic history, and adaptive potential in one of the world’s most geographically restricted felids.

Beyond its immediate conservation relevance, the Nimr1 assembly expands the genomic representation of the *Panthera* radiation and demonstrates the utility of PacBio HiFi sequencing for generating high-quality genomes from critically endangered taxa with limited sample availability. As genomic tools become increasingly central to evidence-based wildlife management, resources such as Nimr1 will be indispensable for guiding breeding decisions, detecting inbreeding depression, and ultimately supporting the long-term recovery of the Arabian leopard.

## Figures and Tables

**Table 1:** Assembly statistics and BUSCO completeness assessment of five *Panthera pardus* genome assemblies evaluated using the carnivora_odb12 lineage dataset (n=13,727 BUSCOs) in BUSCO v6.0.0.

Metric	GCA_038088395.1	GCA_024362965.1	GCA_024362865.1	GCA_001857705.1	GCA_024363415.1
**(A) Assembly Statistics**					
Species/Subspecies	*P. p. nimr*	*P. pardus*	*P. pardus*	*P. pardus*	*P. pardus*
Assembly name	Nimr1	ASM2436296v1	leopard2_amari	PanPar1.0	ASM2436341v1
Number of scaffolds	-	381	389	50,376	7265
Number of contigs	94	716	675	164,952	7277
Total length (Gb)	2.43	2.44	2.44	2.58	2.28
Scaffold N50 (Mbp)	-	126	158	21	1
Contig N50	62 Mbp	22 Mbp	25 Mbp	39 kbp	1 Mbp
Percent gaps (%)	0.000	0.007	0.006	3.846	0.000
**(B) BUSCO Completeness Scores** (carnivora_odb12; n=13,727)					
Complete—C (%)	**99.4**	**99.4**	99.3	98.9	87.8
Single-copy—S (%)	99.1	99.1	99.1	98.4	86.6
Duplicated—D (%)	0.3	0.3	0.3	0.5	1.2
Fragmented—F (%)	0.3	0.3	0.3	0.5	2.3
Missing—M (%)	0.3	0.4	0.4	0.5	9.9
Complete BUSCOs (*n*)	13,643	13,638	13,634	13,581	12,047
Single-copy (*n*)	13,600	13,600	13,599	13,514	11,884
Duplicated (*n*)	43	38	35	67	163
Fragmented BUSCOs (*n*)	38	38	42	73	316
Missing BUSCOs (*n*)	46	51	51	73	1364

C = Complete; S = Single-copy; D = Duplicated; F = Fragmented; M = Missing. BUSCO v6.0.0 was run in euk_genome_min mode using miniprot as the gene predictor and the carnivora_odb12 lineage dataset (created: 1 July 2025; 10 reference genomes). Assembly references: Nimr1 (this study); ASM2436296v1, leopard2_amari, and ASM2436341v1 [14]; PanPar1.0 [16].

## Data Availability

The nuclear genome assembly of *P. p. nimr* (Nimr1) generated in this study is openly available in NCBI GenBank under accession number GCA_038088395.1, subsequently updated to GCA_038088395.2 on 6 May 2026 to incorporate genome annotation information (https://www.ncbi.nlm.nih.gov/datasets/genome/GCA_038088395.2/, accessed on 7 July 2026). Genome annotation data are accessible via the NCBI Gene database (https://www.ncbi.nlm.nih.gov/datasets/gene/GCA_038088395.2/, accessed on 7 July 2026). Raw HiFi sequencing reads are deposited in the NCBI Sequence Read Archive under BioProject PRJNA1091853, SRA accession SRX27330393, and BioSample SAMN40614674, making all raw and assembled data fully accessible and reproducible.

## Supplementary Materials

The following supporting information can be downloaded at: https://www.mdpi.com/article/10.3390/ijms27146115/s1.
