# Supplementary material for: First Genome Assembly of the Critically Endangered Arabian Leopard (Panthera pardus nimr)
Source: Int J Mol Sci. 2026 Jul 8;27(14):6115. doi: 10.3390/ijms27146115 (PMC13409816; doi:10.3390/ijms27146115)
Supplement: Supplementary file 1 [file ijms-27-06115-s001.zip › ijms-4343217-supplementary.pdf]

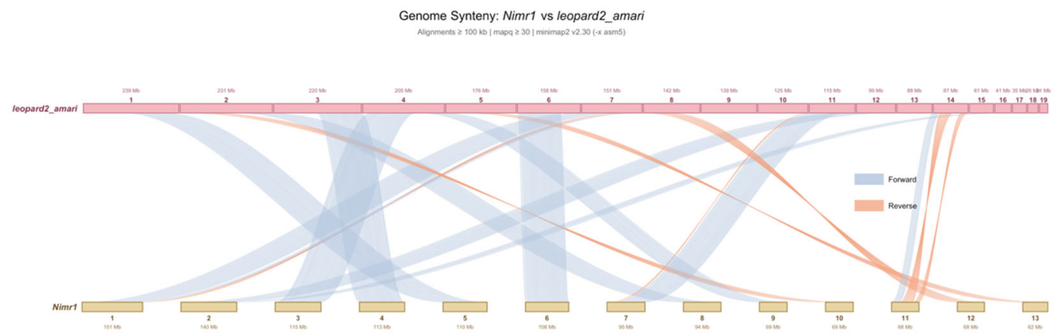

Figure S1. Whole-genome synteny ribbon plot between Nimr1 (*P. p. nimr*) and the Hi-C–scaffolded leopard reference leopard2_amari (GCA_024362865.1), generated using minimap2 v2.30 (-x asm5; alignments ≥ 100 kb, mapping quality ≥ 30). Blue ribbons indicate forward-strand collinearity; orange ribbons indicate reverse-complement (inverted) alignments. The conserved collinearity observed against this second, independently assembled reference is consistent with that seen against ASM2436296v1 (Figure 1C).

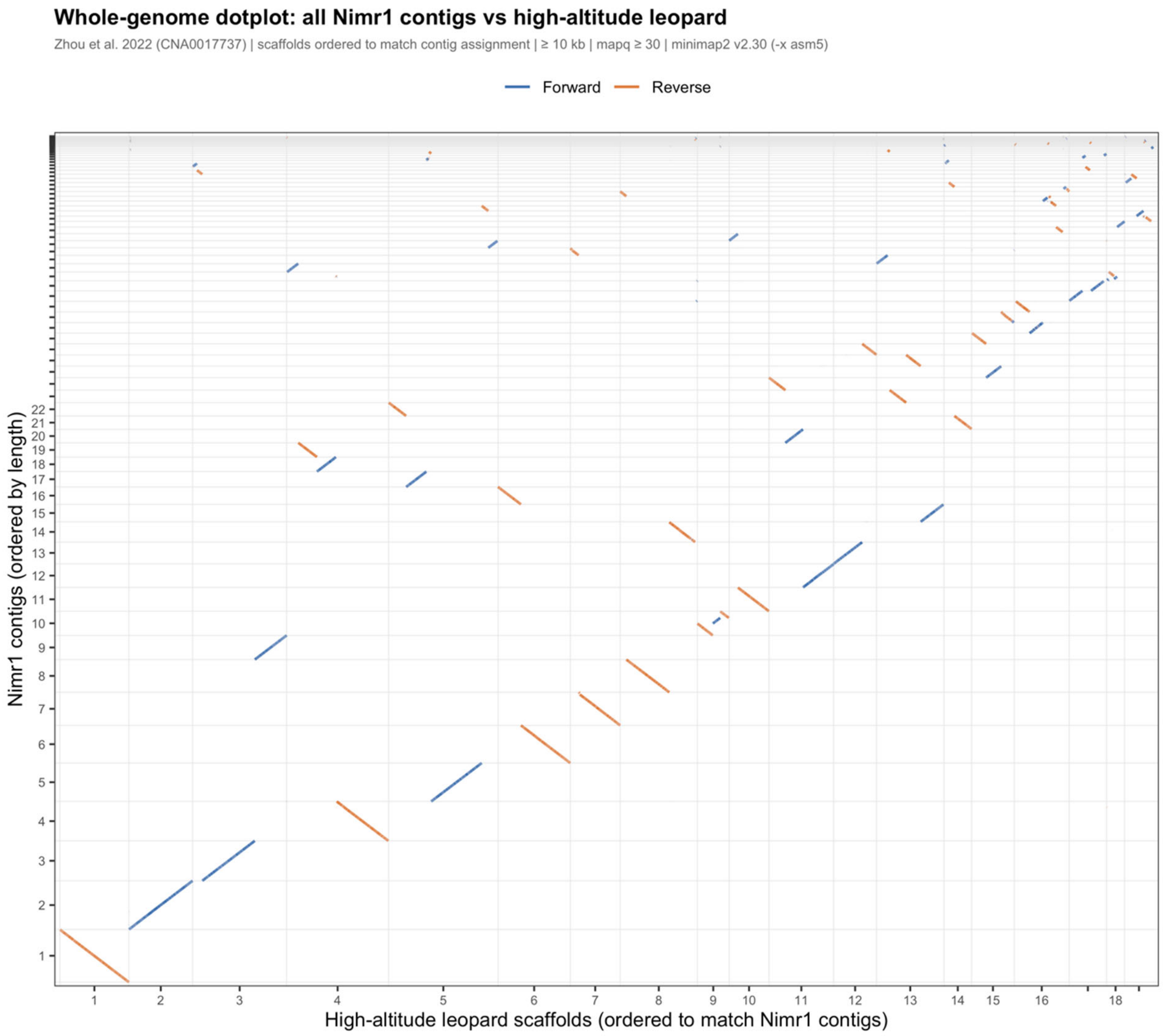

Figure S2. Whole-genome dotplot of all aligning Nimr1 contigs (alignments ≥ 10 kb, mapping quality ≥ 30; minimap2 v2.30, -x asm5) against the chromosome-level high-altitude leopard assembly (Zhou et al. 2022; CNGBdb CNA0017737). Reference scaffolds are ordered to match the Nimr1 contig to which each is primarily assigned, so that a one-to-one correspondence appears along the main diagonal. Blue segments indicate forward-strand (collinear) alignments and orange segments reverse-complement (inverted) alignments. Each chromosome-scale Nimr1 contig aligns predominantly as a single diagonal block to one reference scaffold, confirming that the collinearity observed against the African leopard references is reproduced against this independent high-altitude leopard assembly.

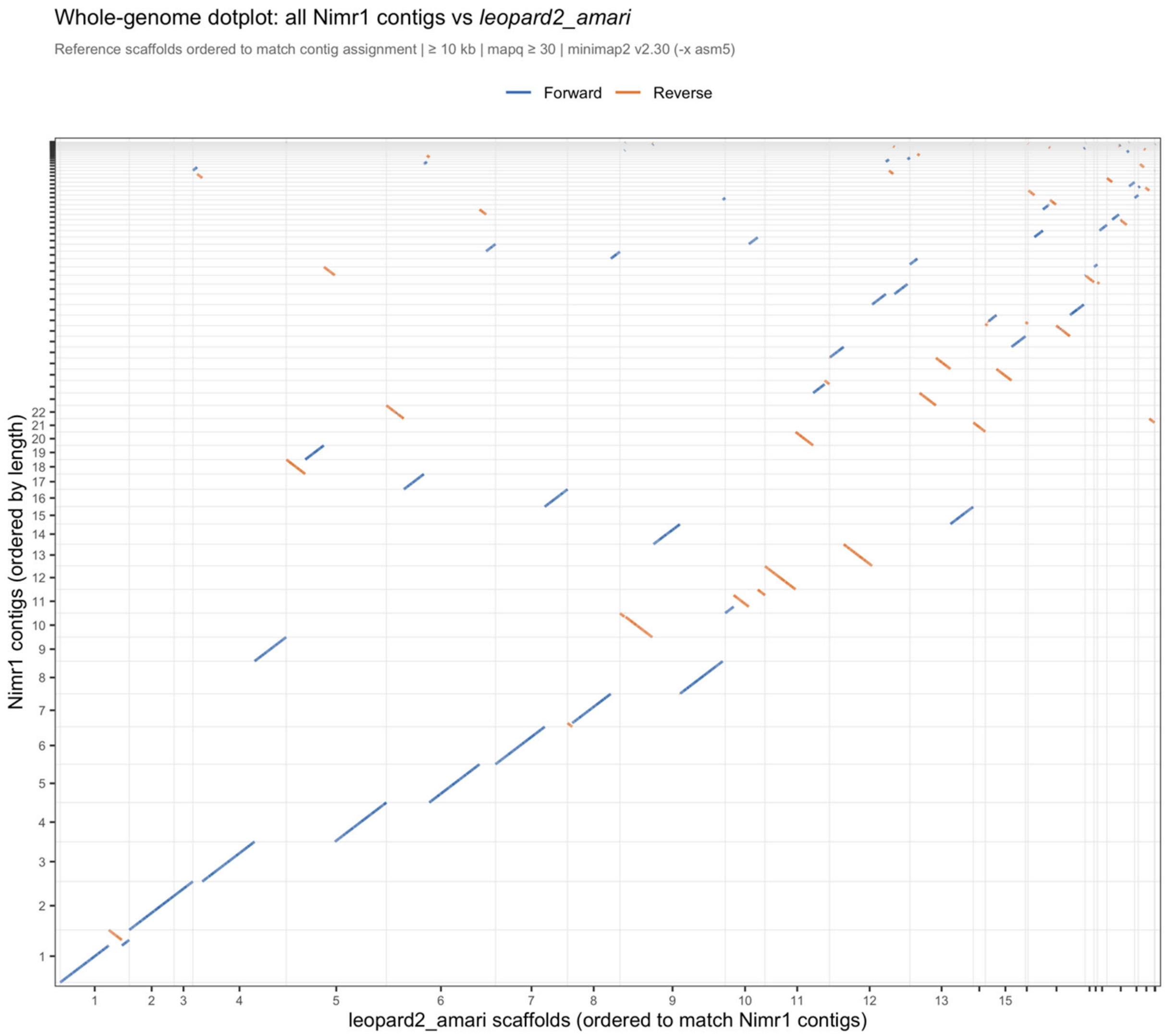

Figure S3. Whole-genome dotplot of all aligning Nimr1 contigs (n = 82; alignments ≥ 10 kb, mapping quality ≥ 30; minimap2 v2.30, -x asm5) against the chromosome-level leopard2_amari assembly (GCA_024362865.1). Reference scaffolds are ordered to match the Nimr1 contig to which each is primarily assigned, so that a one-to-one correspondence appears along the main diagonal. Blue segments indicate forward-strand (collinear) alignments and orange segments indicate reverse-complement (inverted) alignments. Each chromosome-scale Nimr1 contig aligns predominantly as a single diagonal block to one reference scaffold, with cross-chromosomal alignments restricted to a small number of short contigs, confirming that the contigs map uniquely to single chromosomes.

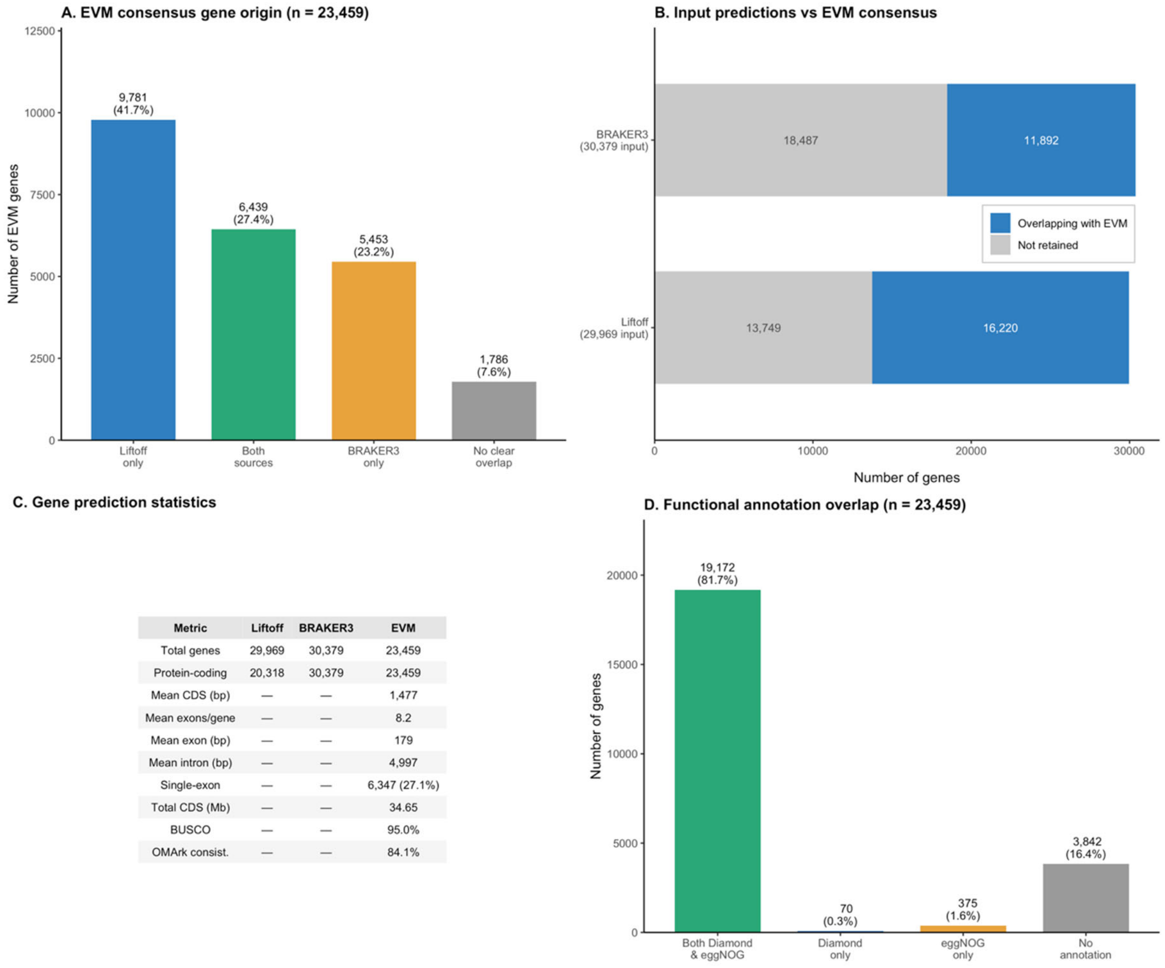

Metric	Liftoff	BRAKER3	EVM
Total genes	29,969	30,379	23,459
Protein-coding	20,318	30,379	23,459
Mean CDS (bp)	—	—	1,477
Mean exons/gene	—	—	8.2
Mean exon (bp)	—	—	179
Mean intron (bp)	—	—	4,997
Single-exon	—	—	6,347 (27.1%)
Total CDS (Mb)	—	—	34.65
BUSCO	—	—	95.0%
OMArk consist.	—	—	84.1%

Figure S4. Evidence-source contribution to the EvidenceModeler (EVM) consensus gene set and functional-annotation overlap for the Nimr1 genome. (A) Origin of the 23,459 EVM consensus genes by structural-evidence support: both Liftoff and BRAKER3 (6,439; 27.4%), Liftoff only (9,781; 41.7%), BRAKER3 only (5,453; 23.2%), and no clear overlap at the ≥50% reciprocal threshold (1,786; 7.6%). (B) Input gene predictions from Liftoff (29,969) and BRAKER3 (30,379) and the number from each source overlapping the EVM consensus (16,220 and 11,892, respectively). (C) Structural and quality-assessment statistics for the EVM consensus annotation. (D) Functional-annotation overlap: genes annotated by both Diamond/Swiss-Prot and eggNOG-mapper (19,172; 81.7%), by Diamond/Swiss-Prot only (70; 0.3%), by eggNOG-mapper only (375; 1.6%), or by neither (3,842; 16.4%).

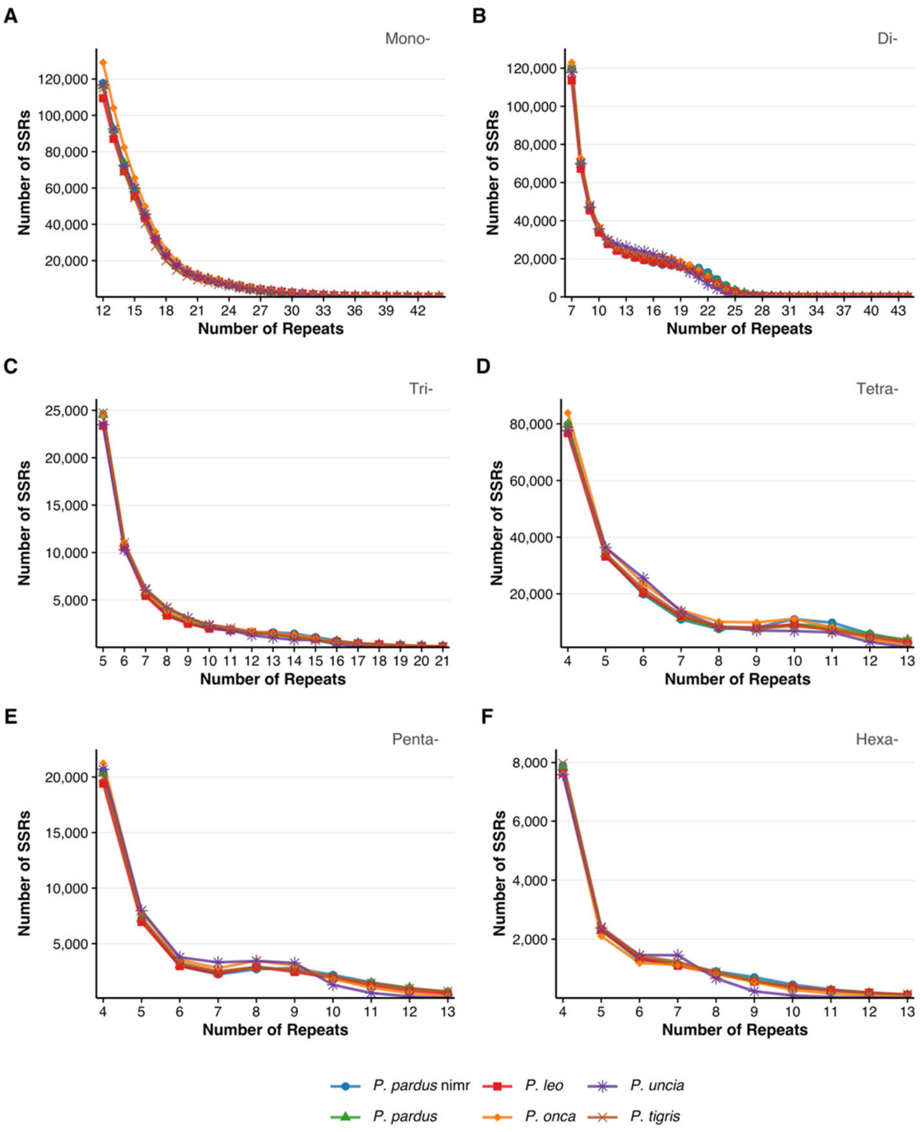

Figure S5. Distribution of SSR repeat number across six *Panthera* species genomes. The number of perfect simple sequence repeats (SSRs) is plotted as a function of repeat number for each of six repeat unit classes: (A) mononucleotide (repeat numbers 12–44), (B) dinucleotide (7–44), (C) trinucleotide (5–21), (D) tetranucleotide (4–13), (E) pentanucleotide (4–13), and (F) hexanucleotide (4–13). Each line represents one species: Arabian leopard (*P. pardus nimr*), leopard (*P. pardus*), lion (*P. leo*), jaguar (P. onca), snow leopard (*P. uncia*), and tiger (*P. tigris*). In all panels, SSR abundance declines expo- nentially with increasing repeat number, reflecting the genome-wide prevalence of shorter tandem repeats. The six species show broadly conserved repeat number distributions across all SSR classes, consistent with their shared evolutionary history within the genus *Panthera*.

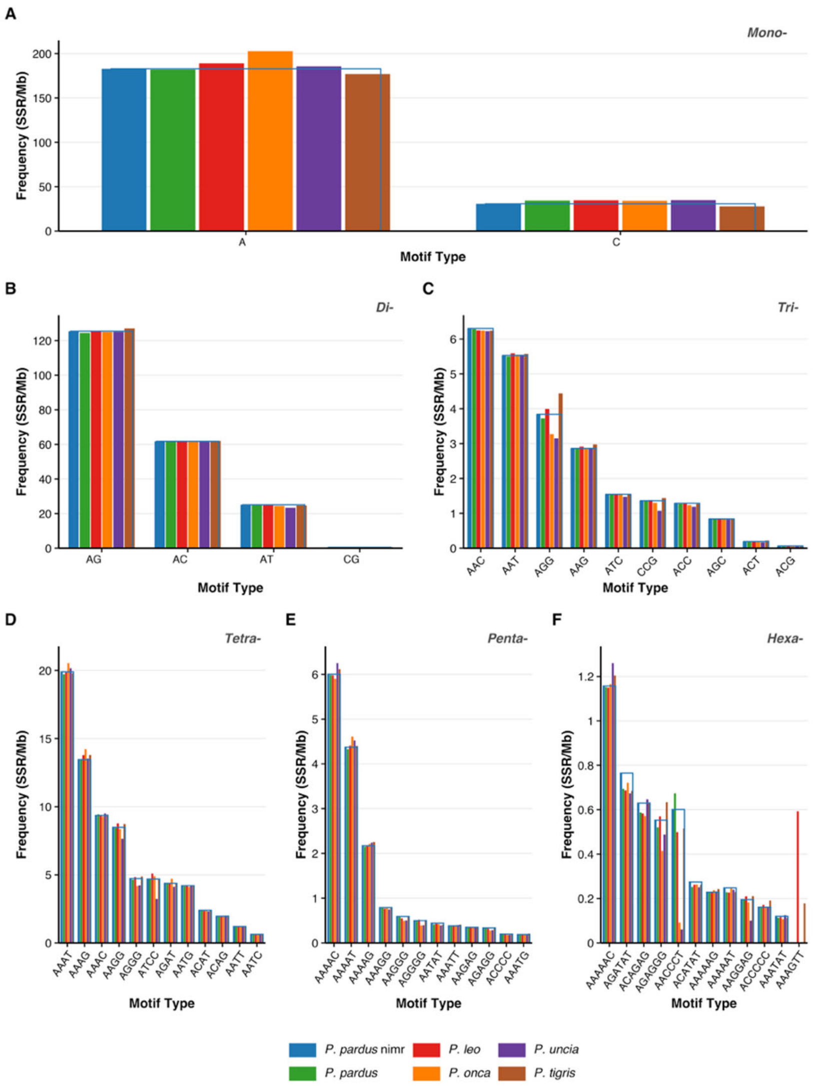

Figure S6. Frequency distribution of SSR motif classes across six *Panthera* species genomes. The fre- quency (SSR/Mb) of each individual sequence motif is shown for six repeat unit classes: (A) mononu- cleotide, (B) dinucleotide, (C) trinucleotide, (D) tetranucleotide, (E) pentanucleotide, and (F) hexanu- cleotide. Each bar group represents a distinct sequence motif, and bars within each group correspond to the six species: Arabian leopard (*P. pardus nimr*), leopard (*P. pardus*), lion (*P. leo*), jaguar (*P. onca*), snow leopard (*P. uncia*), and tiger (*P. tigris*). Frequencies were normalised to the assembled genome size of each species. Note that the y-axis scale differs between panels, reflecting the large differences in overall SSR abundance across repeat unit classes. All six species show broadly similar motif frequency profiles within each repeat class, with a small number of dominant motifs accounting for the majority of SSR content in each category.

Table S1. Reference-guided contig-anchoring summary for the Arabian leopard assembly Nimr1 against the chromosome-level leopard2_amari reference (GCA_024362865.1). For each Nimr1 contig with confident whole-genome alignments (≥ 10 kb, mapping quality ≥ 30; minimap2 v2.30, -x asm5), the assigned reference chromosome (the scaffold receiving the largest share of aligned sequence), the percentage of the contig's aligned sequence directed to that chromosome, and the dominant alignment orientation are listed. Of the 82 contigs with confident alignments, 72 (88%) anchored uniquely to a single reference chromosome (≥ 85% of aligned sequence); the remaining 10 contigs (shaded) fell below this threshold and are predominantly short, likely reflecting repetitive content, reference-scaffold boundaries, or genuine structural differences rather than misassembly.

Nimr1 contig	Length (Mb)	Assigned chromosome	% to assigned	Orientation
JBBMMB010000004.1	151.45	JANFAN010000019.1	99.6	forward
JBBMMB010000002.1	139.72	JANFAN010000008.1	70.6	forward
JBBMMB010000021.1	114.77	JANFAN010000007.1	100	forward
JBBMMB010000019.1	113.25	JANFAN010000001.1	99.8	forward
JBBMMB010000027.1	110.31	JANFAN010000018.1	100	forward
JBBMMB010000029.1	108.16	JANFAN010000017.1	100	forward
JBBMMB010000013.1	95.09	JANFAN010000006.1	100	forward
JBBMMB010000009.1	94.03	JANFAN010000002.1	100	forward
JBBMMB010000032.1	69.46	JANFAN010000007.1	100	forward
JBBMMB010000018.1	69.33	JANFAN010000002.1	99.8	reverse
JBBMMB010000045.1	68.48	JANFAN010000010.1	99.5	reverse
JBBMMB010000016.1	67.87	JANFAN010000011.1	98.5	reverse
JBBMMB010000010.1	62.47	JANFAN010000022.1	99.5	reverse
JBBMMB010000017.1	58.32	JANFAN010000002.1	98.9	forward
JBBMMB010000030.1	50.04	JANFAN010000005.1	99.2	forward
JBBMMB010000007.1	49.89	JANFAN010000017.1	100	forward

Nimr1 contig	Length (Mb)	Assigned chromosome	% to assigned	Orientation
JBBMMB010000008.1	44.38	JANFAN010000018.1	99.5	forward
JBBMMB010000012.1	41.61	JANFAN010000001.1	99.4	reverse
JBBMMB010000020.1	40.88	JANFAN010000001.1	100	forward
JBBMMB010000040.1	38.71	JANFAN010000011.1	100	reverse
JBBMMB010000046.1	38.56	JANFAN010000021.1	68.8	reverse
JBBMMB010000026.1	38.11	JANFAN010000018.1	100	reverse
JBBMMB010000049.1	36.02	JANFAN010000005.1	99.9	reverse
JBBMMB010000022.1	35.76	JANFAN010000011.1	100	forward
JBBMMB010000038.1	33.37	JANFAN010000016.1	99.1	reverse
JBBMMB010000024.1	31.92	JANFAN010000005.1	100	reverse
JBBMMB010000014.1	31.89	JANFAN010000022.1	99	forward
JBBMMB010000028.1	30.66	JANFAN010000016.1	100	forward
JBBMMB010000023.1	30.58	JANFAN010000023.1	98.5	reverse
JBBMMB010000003.1	30.51	JANFAN010000016.1	81.7	forward
JBBMMB010000058.1	30.39	JANFAN010000023.1	100	forward
JBBMMB010000037.1	30.26	JANFAN010000022.1	100	forward
JBBMMB010000034.1	28.71	JANFAN010000022.1	99.9	forward
JBBMMB010000006.1	25.34	JANFAN010000012.1	78.9	reverse
JBBMMB010000035.1	24.15	JANFAN010000001.1	100	reverse
JBBMMB010000001.1	23.92	JANFAN010000005.1	69.7	forward
JBBMMB010000015.1	21.44	JANFAN010000006.1	92.4	forward
JBBMMB010000011.1	20.53	JANFAN010000018.1	100	forward
JBBMMB010000050.1	20.32	JANFAN010000010.1	97.2	forward

Nimr1 contig	Length (Mb)	Assigned chromosome	% to assigned	Orientation
JBBMMB010000039.1	18.54	JANFAN010000023.1	100	forward
JBBMMB010000063.1	16.64	JANFAN010000013.1	98.7	forward
JBBMMB010000051.1	15.09	JANFAN010000014.1	94.9	reverse
JBBMMB010000061.1	14.93	JANFAN010000014.1	100	forward
JBBMMB010000062.1	14.39	JANFAN010000018.1	100	reverse
JBBMMB010000041.1	13.96	JANFAN010000023.1	94.8	forward
JBBMMB010000036.1	13.85	JANFAN010000023.1	96.3	reverse
JBBMMB010000031.1	13.63	JANFAN010000003.1	59.9	forward
JBBMMB010000005.1	13.27	JANFAN010000023.1	100	reverse
JBBMMB010000044.1	12.13	JANFAN010000020.1	100	reverse
JBBMMB010000059.1	12.13	JANFAN010000014.1	98.7	forward
JBBMMB010000055.1	12.02	JANFAN010000014.1	95.7	reverse
JBBMMB010000056.1	11.37	JANFAN010000007.1	100	reverse
JBBMMB010000033.1	9.65	JANFAN010000022.1	100	reverse
JBBMMB010000052.1	9.57	JANFAN010000007.1	94	forward
JBBMMB010000066.1	9.07	JANFAN010000020.1	100	reverse
JBBMMB010000070.1	7	JANFAN010000018.1	93.8	forward
JBBMMB010000043.1	6.75	JANFAN010000022.1	100	forward
JBBMMB010000067.1	6.13	JANFAN010000022.1	90.8	forward
JBBMMB010000025.1	5.17	JANFAN010000018.1	100	reverse
JBBMMB010000057.1	4.68	JANFAN010000005.1	100	reverse
JBBMMB010000053.1	4.52	JANFAN010000023.1	100	forward
JBBMMB010000048.1	4.47	JANFAN010000014.1	100	forward

Nimr1 contig	Length (Mb)	Assigned chromosome	% to assigned	Orientation
JBBMMB010000042.1	3.01	JANFAN010000002.1	53.9	forward
JBBMMB010000060.1	2.75	JANFAN010000020.1	100	reverse
JBBMMB010000073.1	2.57	JANFAN010000023.1	92.4	forward
JBBMMB010000078.1	2.51	JANFAN010000023.1	96.2	reverse
JBBMMB010000072.1	2.33	JANFAN010000022.1	100	reverse
JBBMMB010000064.1	2.11	JANFAN010000014.1	100	reverse
JBBMMB010000047.1	1.69	JANFAN010000014.1	100	forward
JBBMMB010000065.1	1.6	JANFAN010000002.1	100	forward
JBBMMB010000079.1	1.56	JANFAN010000316.1	77.9	forward
JBBMMB010000082.1	1.37	JANFAN010000023.1	71.4	reverse
JBBMMB010000068.1	1.09	JANFAN010000002.1	99	reverse
JBBMMB010000081.1	0.6	JANFAN010000012.1	100	forward
JBBMMB010000075.1	0.55	JANFAN010000002.1	100	forward
JBBMMB010000087.1	0.44	JANFAN010000001.1	77.7	forward
JBBMMB010000084.1	0.4	JANFAN010000014.1	100	forward
JBBMMB010000080.1	0.34	JANFAN010000023.1	100	forward
JBBMMB010000074.1	0.33	JANFAN010000001.1	100	reverse
JBBMMB010000086.1	0.26	JANFAN010000002.1	100	reverse
JBBMMB010000088.1	0.26	JANFAN010000014.1	100	forward
JBBMMB010000090.1	0.06	JANFAN010000369.1	100	reverse

Shaded rows indicate contigs flagged as conflicting (< 85% of aligned sequence to a single reference chromosome).

Table S2. Haplotype assembly metrics and assembly quality-control statistics for the Arabian leopard (*Panthera pardus nimr*) genome Nimr1. Values are shown for the published primary assembly (GCA_038088395.2; 94 contigs after adaptor and contaminant filtering of the 273-contig raw HiFiasm output) and for the two HiFiasm haplotype assemblies (haplotype 1 and haplotype 2). Consensus quality (QV) and k-mer completeness were computed with Merqury from a 21-mer database of the HiFi reads; mapping rate, coverage, variant density, homozygous blocks, and contamination were assessed on the primary assembly. The near-zero F(ROH) is reported as a preliminary estimate, as discussed in the main text.

Metric	Primary assembly	Haplotype 1	Haplotype 2
Total length (Gb)	2.43	2.39	2.33
Number of contigs	94	558	521
Contig N50 (Mb)	62.4	49.9	67.1
L50	13	14	12
Largest contig (Mb)	151.4	151.4	148.3
GC content (%)	41.73	41.76	41.70
Merqury QV	49.5	49.3	48.6
K-mer completeness (%)	98.03	96.43	95.27
Mapping rate (%)	99.99	—	—
Mean coverage (×)	66.1 (σ 16.6)	—	—
Heterozygosity (per kb)	0.30	—	—
SNP density (per kb)	0.19	—	—
Indel density (per kb)	0.12	—	—
Homozygous blocks	3 (F(ROH) ≈ 0)	—	—
Contamination (NCBI FCS)	1 adaptor (33 bp)	—	—

—, not applicable. QV, consensus quality value (Phred scale). F(ROH) estimated from three short homozygous blocks (14,554 bp total).

Table S3. Gene-prediction and structural annotation statistics for the Nimr1 genome. Gene counts are shown for the two input evidence sources (Liftoff homology transfer and BRAKER3 ab initio prediction) and for the final EvidenceModeler (EVM) consensus, together with structural metrics and quality-assessment scores for the consensus annotation. The consensus gene count (23,459) is lower than either input set, reflecting consolidation of redundant and conflicting models rather than inflation.

Category	Metric	Value	Notes
**Liftoff input**	Total genes	29,969	*From African leopard reference*
	Protein-coding genes	20,318	
	Unmapped features	579	
**BRAKER3 input**	Total genes	30,379	*OrthoDB v11 evidence*
	Protein sequences	32,808	*Multiple isoforms*
**EVM consensus**	Protein-coding genes	23,459	*Liftoff weight 5; BRAKER3 weight 2*
	Mean exons per gene	8.2	
	Mean CDS length (bp)	1,477	
	Mean exon length (bp)	179	
	Mean intron length (bp)	4,997	
	Single-exon genes	6,347 (27.1%)	
	Total CDS length (Mb)	34.65	
**Quality assessment**	BUSCO completeness	95.0%	*carnivora_odb10*
	OMArk consistency	84.1%	*Panthera lineage*
	OMArk contamination	0.0%	
